# Work-to-Family Conflict and Children’s Problems with School, Friends, and Health: Household Economic Conditions and Couple Relationship Quality as Contingencies

**DOI:** 10.1177/0192513X211026953

**Published:** 2021-06-25

**Authors:** Lei Chai, Scott Schieman

**Affiliations:** 1 Department of Sociology, 7938 University of Toronto, Toronto, ON, Canada

**Keywords:** work-to-family conflict, children’s problems, couple relationship quality, economic conditions, stress amplification theory, ecological model of human development

## Abstract

What is the relationship between work-to-family conflict (WFC) and children’s problems with school, friends, and health? And does that association depend on household economic conditions and couple relationship quality? Using four waves of longitudinal data from the Canadian Work, Stress, and Heath Study (2011–2017), the present study finds that—overall—both fathers’ and mothers’ levels of WFC are associated with elevated levels of children’s problems over time. However, we also discover that household income and spousal disputes moderate this focal relationship—and they do so differently for mothers and fathers. First, the positive association between WFC and children’s problems is stronger for mothers (but not fathers) in households with lower income. Second, the positive association between WFC and children’s problems is stronger for fathers (but not mothers) who report more frequent disputes with their spouse. We discuss the implications of these patterns for current theorizing about stress amplification dynamics and situate that discussion within broader ideas in the ecological model of human development.

## Introduction

Research on work-to-family conflict (hereafter “WFC”) has proliferated in recent years. Work-to-family conflict is a chronic inter-role stressor that captures the process in which the work role detracts from the time, attention, and performance of the family role (Badawy & Schieman, 2020b). Prior research shows that over 60% of Canadian workers report moderate or high levels of WFC—and this has increased over time (Duxbury & Higgins, 2013). The incompatibility between work and family roles has detrimental consequences for health as research shows that WFC is associated with elevated psychological distress or physical symptoms (Badawy & Schieman, 2020b). More recently, some scholars have sought to extend the scope of research to understand the impact on children’s well-being (e.g., Matias et al., 2017; Yucel & Latshaw, 2020). Due to the increasing prevalence of WFC, parents in dual-earner couples face difficulties with competing demands between work and family domains. Therefore, understanding the link between WFC and children’s outcomes has practical implications. For instance, workplace policies that help parents reduce role strains could improve their own role functioning and their children’s well-being.

Given that social stressors often coexist with others (Pearlin, 1989), in addition to exploring the potential consequences attached to WFC, a growing literature has also sought to document the impact of other stressors commonly faced by dual-earner couples on children’s outcomes. Informed by Pearlin’s (1989) stress process model and Bronfenbrenner’s (1986) ecological model of human development, studies have revealed that household economic conditions (Conger & Conger, 2002; Conger, Conger, & Martin, 2010) and couple relationship quality (Dinh et al., 2017; Vahedi, Krug, Fuller-Tyszkiewicz, & Westrupp, 2018) are negatively associated with children’s outcomes. Given that experiencing multiple stressors simultaneously are often linked to greater health disparities (Young & Schieman, 2012), it is unclear whether the joint experiences of WFC and both adverse household economic conditions and relationship strains are more detrimental to children’s outcomes.

To address this puzzle, we analyze four waves of panel data from a national survey of Canadian workers (2011–2017). We ask three research questions. First, how does WFC predict children’s problems over time? We focus on three common difficulties that children face: school, friends, and health (Badawy & Schieman, 2020a). Second, we answer recent calls from scholars that more research is needed on the ways that family-related conditions moderate the association between WFC and children’s problems (Yucel & Latshaw, 2020). For reasons described below, we focus on the following household and couple characteristics as moderators: (1) income, (2) financial strain, (3) marital dissatisfaction, and (4) spousal disputes. Third, recognizing that mothers and fathers might diverge in their responses to these moderating conditions, we assess potential gender differences in these dynamics.

The present study makes three contributions. First, to the best of our knowledge, only one study by Yucel and Latshaw (2020) has assessed the extent to which family-related conditions *moderate* the association between WFC and children’s problems. We seek to build on Yucel and Latshaw’s study by including the subjective experience of financial strain alongside the objective measure of household income. Moreover, we also assess two relational dynamics as moderators: marital dissatisfaction and spousal disputes. Our second contribution extends Yucel and Latshaw’s cross-sectional study design by testing random effects models using a longitudinal design—this allows us to describe between-individual level changes over time. Our third contribution is more theoretical: We seek to integrate the ecological model of human development (Bronfenbrenner, 1986) and stress amplification theory (Young & Schieman, 2012) to understand the factors that moderate the link between WFC and children’s problems.

## Literature Review and Hypotheses

### WFC and Children’s Problems

Bronfenbrenner’s (1986) ecological model of human development is a theoretical framework to explain the association between WFC and children’s outcomes. Past research that applies the ecological model has underscored the ways in which the interactions of four key systems shape children’s development, including the microsystem, mesosystem, exosystem, and macrosystem. Rosa and Tudge (2013) define each of these systems: The microsystem refers to “the most proximal setting, with particular physical characteristics, in which a person is situated” (p. 246). Interaction with parents plays a significant role in shaping children’s outcomes; this is referred to as children’s microsystem. Second, the mesosystem refers to “the relations among two or more microsystems in which the developing person actively participate” (p. 246). Both home and school represent a microsystem that shapes children’s development. However, the further connections between these two different agents comprise children’s mesosystem. Third, the exosystem refers to “an ecological setting in which the developing person of interest is not situated, and thus does not participate actively within it, but nonetheless experiences its influence” (p. 246). The parents’ workplace can be viewed as children’s exosystem where children are not actively engaged in this system, and it nevertheless has indirect effects on children’s development. Parents who work long hours might limit the amount of time spent with children, which interrupts family routines, and adversely affects family functioning. And fourth, the macrosystem emphasizes “the institutional systems of a culture or subculture, such as the economic, social, education, legal, and political systems” (p. 246). Applying these ideas about different systems in the present study, WFC represents a process where parents’ job demands spill over from the work microsystem into the family microsystem (i.e., parents’ mesosystem) (Eby, Casper, Lockwood, Bordeaux, & Brinley, 2005). When parents experience difficulties with excessive work demands—a characteristic in children’s exosystem—they might not be able to fully monitor and fulfill the needs of children, which can negatively affect parental role functioning. This has implications for the quality of children’s microsystem (Bronfenbrenner, 1986), which, in turn, negatively influences children’s well-being (Chee, Conger, & Elder, 2009).

Recent studies support Bronfenbrenner’s (1986) theoretical propositions. Using five waves of Australian data, Dinh et al. (2017) found that increases in WFC were associated with higher levels of children’s problems (e.g., emotional symptoms, conduct problems, hyperactivity/inattention, and peer relationship problems). One potential limitation of that study is that it combines two distinct directions of work–family conflict—work-to-family conflict and family-to-work conflict (FWC)—into one single measure. Conceptual specifications and prior evidence show that WFC and FWC are weakly to moderately correlated (Bellavia & Frone, 2005), suggesting that each direction has different antecedents and, in some cases, different consequences (French, Dumani, Allen, & Shockley, 2018). Work–family scholarship therefore encourages the assessment of the distinct influences of these two dimensions (French et al., 2018). Given that children’s problems are more likely to be a cause rather than an outcome of FWC (Yucel & Latshaw, 2020), we focus solely on WFC as a potential determinant of children’s problems. Here, we build upon two studies that assess WFC’s link to children’s problems. Matias et al. (2017) found that WFC was associated with higher levels of children’s emotional lability and lower levels of children’s emotional regulation. Likewise, Yucel and Latshaw (2020) found that WFC was associated with higher levels of children’s emotional problems. Taken together, theoretical ideas and evidence motivate the following (**Hypothesis 1**): WFC will be associated with higher levels of children’s problems.

### Potential Moderators: Household Economic Conditions and Couple Relationship Quality

The stress process model guides our focus on family-related conditions—both economic and interpersonal—as potential *moderators* (Pearlin, 1989). Prior research in the stress process tradition underscores that stressors coexist and sometimes interact in a multiplicative fashion (Avison & Comeau, 2013). Recent research refers to this dynamic as “stress amplification” (Badawy & Schieman, 2020a). Parents face greater challenges when they experience multiple stressors simultaneously because this undermines coping capacity (Young & Schieman, 2012). Applied here, stress amplification theory predicts that WFC will be more problematic when parents simultaneously experience other stressors in the family domain.^1^ This implies a multiplicative effect between WFC and both household economic conditions and couple relationship quality.

#### Household Economic Conditions

Prior research has sought to understand the association between household economic conditions and children’s well-being, suggesting a link between adverse economic conditions and parents’ own emotional, behavioral, and role functioning—processes that ultimately shape children’s outcomes like reduced cognitive functioning and increased emotional and behavioral problems (Conger & Conger, 2002; Conger et al., 2010). We build on prior research about the importance of economic conditions as a family stressor to understand its role as a potential moderator. Stress amplification theory predicts that WFC should be more consequential for children’s problems when it *combines with* economic challenges. When parents experience work-role demands that spill over into non-work roles, this might create difficulties for consistent and focused parenting practices. Inter-role conflict—especially if it occurs in the context of economic challenges—might amplify strains associated with the parental role and ultimately elevate children’s adverse outcomes.

While prior studies have focused on levels of household income for understanding children’s problems (Reiss, 2013), few have included subjective financial strain. Scholars underscore the importance of distinguishing subjective perceptions of financial strain from objective income measures because of their distinct meanings and individual effects on health (Arber, Fenn, & Meadows, 2014). Financial strain is defined as the perception of having difficulties making ends meet, including paying bills or purchasing food, clothing, and housing (Kahn & Pearlin, 2006). Although household income is correlated with financial strain (Pudrovska et al., 2005), this subjective evaluation represents a potentially relevant stressor on its own—across the socioeconomic spectrum—because individuals with moderate-to-high levels of household income are not immune to financial strain (McCloud & Dwyer, 2011). Taken together, low household income and high financial strain each represent critically important family-related stressors that—when combined with WFC—might impede parents’ ability to adequately perform the parental role. Based on these ideas, we propose the following (**Hypothesis 2**): The positive association between WFC and children’s problems will be stronger among parents in households with low income and high financial strain, respectively.

#### Couple Relationship Quality

The ecological model posits that children’s development is related to interactions among other family members (Bronfenbrenner, 1986). The quality of parents’ relationship is viewed as the center of the system, so it has the potential to profoundly influence family functioning (Easterbrooks & Emde, 1988). As a primary social exchange relationship, spouses are expected to provide emotional, informational, and instrumental forms of social support to each other (Thoits, 2011; Umberson & Thomeer, 2020). Poor relationship quality represents a source of family role strain (Pearlin, 1989). When individuals within couples experience unfavorable feelings about the relationship that can exacerbate other stressors and ultimately foster physical and mental exhaustion (Westman, Etzion, & Danon, 2001). In turn, these relationship troubles have implications for the well-being and development of their children (Goldberg & Carlson, 2014). For instance, chronic exposure to marital discord and strife can negatively and directly shape children’s emotional and behavioral outcomes (Mannering et al., 2011) and indirectly have harmful effects through parenting behaviors and parent–child interactions (Engfer, 1988; Erel & Burman, 1995).

While these ideas trace a direct line from interpersonal strain in couples to children’s problems, our focus examines how such interpersonal strains in the couple interact with WFC to shape children’s problems. As we described for economic conditions, stress amplification theory once again provides the guiding rationale (Avison & Comeau, 2013; Badawy & Schieman, 2020a; Young & Schieman, 2012). It predicts that poorer relationship quality—as measured by relationship dissatisfaction and spousal disputes—should exacerbate the association between WFC and elevated levels of children’s problems. The multiplicative mechanism is represented in the following (**Hypothesis 3**): The adverse effect of WFC on children’s problems should be even more consequential among parents who experience more relationship dissatisfaction and more frequent disputes with their spouse, respectively.

### Considering Differences between Women and Men

Given that women and men might respond to household economic adversity and relationship strains differently, the aforementioned moderating potentials might further vary across gender. On the one hand, despite significant increases in women’s labor force participation and dual-earner households, being a “breadwinner” tends to remain important to many heterosexual men’s masculine identity (Thébaud, 2010). According to the “doing gender” perspective (Geist & Ruppanner, 2018), men are more likely to avoid performing female-oriented domestic tasks when they earn less than their partners (Thébaud, 2010). The same pattern might also apply to households with low income. Given the persistent gender gap in earnings (Graf, Brown, & Patten, 2018), having a low household income might reflect a husband’s lack of financial contribution to the household. Women in this scenario might therefore take on more housework and childcare responsibilities, which undermines their health and well-being (Khawaja & Habib, 2007; Ruppanner, Brandén, & Turunen, 2018). Moreover, prior research suggests that women in households with greater economic adversity might experience additional stress because of the struggles associated with meeting financial obligations (Thorne, 2010). There is evidence that the division of financial-related tasks appears to be gendered in low-income households, with wives being solely responsible for handling money and debt (e.g., shifting and allocating money or negotiating with debt collectors) because of a lack of support from their husbands. This, in turn, might contribute to elevated emotional stress among wives (Thorne, 2010). Together, these ideas and evidence motivate the following (**Hypothesis 4a**): The adverse moderating effect of household economic conditions in the association between WFC and children’s problems will be stronger for women compared to men.

On the other hand, within the stress process framework, social support has been considered a prominent resource that provides emotional care and comfort, thereby buffering against harmful effects of stressors (Thoits, 2011; Umberson & Thomeer, 2020). According to the gender-role socialization perspective, women are more likely than men to have more extensive social networks because of their greater interpersonal skills (Jung, 2014). In addition to spouses, women often view their friends and other family members as intimate sources of support (Donato, Leon-Perez, Wallston, & Kripalani, 2018). By contrast, masculinity embedded in traditional cultural norms promotes the idea that men should not rely on external resources when dealing with stressful encounters (Bennett, 2007). In addition, given that men often consider their spouses as a primary source of support, they might not disclose personal struggles to their friends or family members (Donato et al., 2018). Together, when dealing with interpersonal strains, women can still derive greater coping efficacy from a variety of nonmarital support compared to men. Collectively, these ideas suggest the following (**Hypothesis 4b**): The adverse moderating effect of couple relationship quality in the association between WFC and children’s problems will be stronger for men compared to women.

## Data and Methods

### Sample

To test the hypotheses outlined above, we analyze data from four waves (2011–2017) of the Canadian Work, Stress, and Health study, a national longitudinal study of the Canadian labor force. To be eligible, individuals had to be (1) residing in Canada; (2) 18 years of age or older; (3) currently working at a paid job or operated an income-producing business; (4) employed in the civilian labor force; and (5) live in a non-institutional residence. In households with more than one eligible person, we used the “next birthday” method to randomly select a participant. Calls were made to a regionally stratified unclustered random probability sample generated by random-digit dial methods (including cell phones). Wave 1 interviews were conducted by telephone between January and August 2011. Subsequent interviews for Waves 2, 3, and 4 were conducted every two years. The final full sample for Wave 1 was 6004, with a response rate of approximately 40%. The number of cases and retention rates for each successive wave of data collection are as follows: Wave 2 *N* = 4423 (73.7% of Wave 1), Wave 3 *N* = 3805 (63.4% of Wave 1 and 86.0% of Wave 2), and Wave 4 *N* = 3378 (56.3% of Wave 1, 76.4% of Wave 2, and 88.8% of Wave 3). We selected partnered individuals who were both employed and had children under age 18 years living in the household. The final sample was 1022 observations (2885 person-years).^2^

### Measures

#### Children’s Problems

We used three items to measure children’s problems (Badawy & Schieman, 2020a). Respondents were asked to report the frequency that their children experienced the following in the past 3 months: “problems at school,” “problems with friends or peers,” and “health problems.” Responses included: “very often" (1), “often" (2), “sometimes" (3), “rarely" (4), and “never" (5). Responses were reverse-coded and averaged to create the index such that higher scores indicated more frequent children’s problems (*α*_w1_ = .62).

#### Work-to-Family Conflict

We used four items to measure WFC (Badawy & Schieman, 2020b). The items asked respondents how often in the past 3 months they had experienced the following: “not had enough time for the important people in your life because of your job,” “not have the energy to do things with the important people in your life because of your job,” “work kept you from doing as good a job at home as you could,” and “job kept you from concentrating on important things in your family or personal life.” Response choices were “very often" (1), “often” (2), “sometimes” (3), “rarely” (4), and “never” (5). We reversed-coded and averaged items such that higher scores indicated more WFC (α_w1_ = .90).

#### Household income

was measured in six categories, including “$25,000 or less” (1), “$25,001 to $50,000” (2), “$50,001 to $75,000” (3), “$75,001 to $100,000” (4), “$100,001 to $125,000” (5), and “$125,001 or more” (6). Given the small cell sizes in the lowest categories, we further recoded the variable into three categories (1 = “$75,000 or less,” 2 = “$75,001 to $125,000,” and 3 = “$125,001 or more”).

#### Financial Strain

We used three items to measure financial strain. The first two items asked how often in the past year study participants had “trouble paying the bills” and “not have enough money to buy food, clothes or things household needed.” Response choices were “very often” (1), “often” (2), “sometimes” (3), “rarely” (4), and “never” (5). The third item asked: “how do your finances usually work out by the end of the month?” Response choices included “a lot of money left over” (1), “a little money left over” (2), “just enough to make ends meet” (3), and “not enough to make ends meet” (4). Similar to recent studies (Chai, Schieman, & Bierman, 2020), we reverse-coded the first two items and then created an index after standardizing all three items (because of different response choices). Higher scores reflected more financial strain (*α*_w1_ = .80).

#### Marital Dissatisfaction

We used three items to measure marital dissatisfaction (Young, Schieman, & Milkie, 2013). The items asked respondents the extent to which they agreed with the following: “I feel very close to my spouse/partner,” “my spouse/partner takes the time to talk over my problems with me,” and “I know that my spouse/partner will always be there for me.” Response choices included “strongly disagree” (1), “somewhat disagree” (2), “somewhat agree” (3), and “strongly agree” (4). We reverse-coded and averaged the items so that higher scores reflected greater marital dissatisfaction (*α*_w1_ = .77).

#### Spousal Disputes

We used one item to measure spousal disputes. Respondents were asked how often they argued with their spouse about housework, finances, or the relationship in the past 3 months. Response choices included “never” (1), “rarely” (2), “sometimes” (3), “often” (4), and “very often” (5). We recoded “often/very often” as “1” and “never/rarely/sometimes” as “0.”

All analyses adjusted for the following variables. *Marital status* was coded as “married” (1) and “cohabiting” (2). *Age of youngest child in the household* was coded as “0–5 years old” (1), “6–11 years old” (2), and “12–18 years old” (3). *Education* was recoded as follows: “less than high school” (1), “high school” (2), “some college” (3), “college” (4), and “postgraduate” (5). *Occupation* was coded in the following categories: “managers” (1), “professionals (2)”, “technical” (3), “sales” (4), “administrative support” (5), “service” (6), and “production” (7). *Respondent’s weekly work hours* were coded in hours. *Spouse’s weekly work hours* were coded in hours. Finally, we included the *survey-year* variable with four categories where the reference group was the first wave (2011). Table 1 presents descriptive statistics of all variables in the analyses.

**Table 1.** Descriptive Statistics of Selected Variables in the Analyses.

	Full Sample		Men		Women	
		Means/%s	*SD*	Means/%s	*SD*	Means/%s	*SD*
Children’s problems	1.84	.66	1.82	.62	1.86	.69
WFC	2.58	.93	2.54	.90	2.60	.94
Household income						
Less than $75,000	15.15%		14.47%		15.60%	
$75,001 to $125,000	34.10%		34.54%		33.82%	
$125,001 or more (REF)	50.75%		50.99%		50.58%	
Financial strain	−.16	.72	−.22	.66	−.12	.75
Couple relationship dissatisfaction	1.36	.53	1.34	.51	1.38	.54
Spousal disputes						
Often/very often	10.29%		8.18%		11.72%	
Never/rarely/sometimes (REF)	89.71%		91.82%		88.28%	
Marital status						
Married (REF)	81.70%		81.83%		81.61%	
Cohabiting	18.30%		18.17%		18.39%	
Age of youngest child, years						
0–5 (REF)	13.41%		14.21%		12.88%	
6–11	35.08%		36.78%		33.93%	
12–18	51.51%		49.01%		53.19%	
Education						
Less than high school	2.84%		5.00%		1.39%	
High school (REF)	11.16%		12.40%		10.32%	
Some college	21.77%		24.55%		19.90%	
College	44.64%		39.79%		47.91%	
Postgraduate	19.58%		18.26%		20.48%	
Occupation						
Managers (REF)	18.34%		22.31%		15.66%	
Professionals	33.93%		29.72%		36.77%	
Technical	16.29%		12.06%		19.14%	
Sales	4.02%		4.22%		3.89%	
Admin support	6.55%		3.70%		8.47%	
Service	9.50%		5.77%		12.01%	
Production	11.37%		22.22%		4.06%	
Respondent’s work hours	39.20	11.81	43.91	10.79	36.03	11.41
Spouse’s work hours	40.90	11.66	35.49	11.21	44.55	10.48
*N* (observations)	1022		409		613	
*N* (person-years)	2885		1161		1724	

*Note*: WFC = work-to-family conflict. “REF” refers to “the reference category” being used in the analyses.

### Analytical Models

We used random effects models to test several models predicting children’s problems. All models included the full set of control variables. In Table 2, Models 1a and 1b tested the direct effect of WFC on children’s problems for men and women, respectively. Next, in Models 2a–5b, we examined whether the association between WFC and children’s problems differed across household income, financial strain, marital dissatisfaction, and spousal disputes. Finally, we used three-way interactions to test gender differences in how household income, financial strain, marital dissatisfaction, and spousal disputes shaped the association between WFC and children’s problems over time. The Hausman test was used to determine whether random effects models were more appropriate than fixed effects models. Each test revealed that fixed effects models would be biased, so we opted for random effects models.

**Table 2.** Random Effects Models Predicting Children’s Problems.

	Model 1a: Fathers	Model 1b: Mothers	Model 2a: Fathers	Model 2b: Mothers	Model 3a: Fathers	Model 3b: Mothers	Model 4a: Fathers	Model 4b: Mothers	Model 5a: Fathers	Model 5b: Mothers
WFC	.104^∗^^∗^^∗^ (.021)	.101^∗^^∗^^∗^ (.019)	.113^∗^^∗^^∗^ (.028)	.055^∗^ (.025)	.105^∗^^∗^^∗^ (.022)	.102∗∗∗ (.019)	.100 (.055)	.163^∗^^∗^^∗^ (.045)	.092^∗^^∗^^∗^ (.022)	.106^∗^^∗^^∗^ (.020)
Household income										
Less than $75,000	.078 (.021)	.113^∗^ (.053)	.229 (.145)	−.310^∗^ (.129)	.078 (.058)	.113^∗^ (.054)	.078 (.058)	.112^∗^ (.053)	.075 (.058)	.111^∗^ (.053)
$75,001-$125,000	−.015 (.041)	−.011 (.037)	−.026 (.111)	−.165 (.097)	−.015 (.041)	−.010 (.037)	−.015 (.041)	−.011 (.037)	−.014 (.041)	−.010 (.037)
$125,001 or more (REF) WFC X household income										
WFC X less than $75,000			−.060 (.052)	.169^∗^^∗^^∗^ (.047)						
WFC X $75,001-$125,000			.005 (.040)	.061 (.035)						
Financial strain	.067^∗^ (.030)	.086^∗^^∗^^∗^ (.025)	.068^∗^ (.030)	.082^∗^^∗^^∗^ (.025)	.063 (.082)	.067 (.063)	.067^∗^ (.030)	.085^∗^^∗^^∗^ (.025)	.064^∗^ (.030)	.086^∗^^∗^^∗^ (.025)
WFC X financial strain					.001 (.027)	.007 (.021)				
Couple relationship dissatisfaction	.077 (.039)	.026 (.034)	.074 (.039)	.025 (.034)	.077 (.039)	.027 (.034)	.068 (.110)	.146 (.086)	.080^∗^ (.039)	.026 (.034)
WFC X couple relationship dissatisfaction							.003 (.039)	−.045 (.030)		
Spousal disputes (1 = very often/often)	.051 (.063)	.011 (.055)	.055 (.063)	.012 (.054)	.051 (.063)	.011 (.055)	.050 (.064)	.019 (.055)	−.282 (.178)	.114 (.154)
WFC X spousal disputes									.116^∗^ (.058)	−.035 (.049)
Intercept	.994	1.181	.984	1.296	.993	1.176	1.005	1.025	1.017	1.175
$Overall\;R^{2}$	.132	.050	.133	.082	.132	.081	.132	.082	.132	.082
*N* (observations)	409	613	409	613	409	613	409	613	409	613
*N* (person-years)	1161	1724	1161	1724	1161	1724	1161	1724	1161	1724

*Note*: WFC = work-to-family conflict. All models include marital status, age of youngest child, education, occupation, respondent’s work hours, spouse's work hours, and survey years. Standard errors are in parentheses.

∗*p* < .05; ∗∗*p* < .01; ∗∗∗*p* < .001.

## Results

The analyses shown in Table 2 estimate the focal association between WFC and children’s problems. In Model 1a, we observe that fathers’ WFC is associated with elevated levels of children’s problems (b = .104, *p* < .001), net of all control variables. Likewise, as shown in Model 1b, mothers’ WFC is also associated with greater children’s problems (b = .101, *p* < .001). These patterns—highly similar for both women and men—support Hypothesis 1. In separate analyses, we tested a two-way interaction term to assess gender differences in the association between WFC and children’s problems. However, we found no evidence of gender differences in association between WFC and children’s problems (results not shown but available upon request).

**Figure 1.**
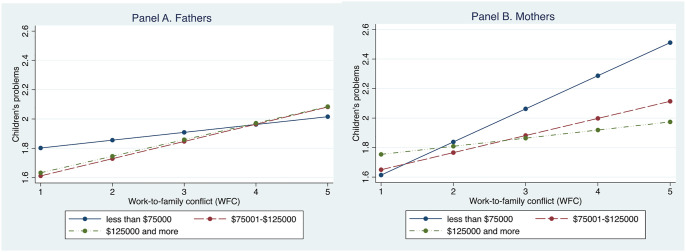
Predicted value in children’s problems by work-to-family conflict and household income for fathers (left) and mothers (right), with all covariates held at their means.

In Models 2a through 5b, respectively, we test whether the positive association between WFC and children’s problems depends on household income, financial strain, couple relationship dissatisfaction, and spousal disputes. In Model 2a, we test household income as a moderator of the association between fathers’ WFC and children’s problems; the results show that household income does not moderate that association. By contrast, in Model 2b, we observe that the positive association between WFC and children’s problems is stronger for mothers with lower household income (b = .169, *p* < .001). We display this interaction effect in Figure 1, with predicted values in children’s problems across WFC and household income for fathers and mothers, respectively. The figure shows that the slope for the link between WFC and children’s problems is steepest among women in households with less than $75,000; that same pattern is not observed among men. These results partially support Hypothesis 2. We also test a three-way interaction term for gender differences in how household income moderates the association between WFC and children’s problems. Those results show that gender differences in the two-way interaction term are statistically significant (b = .228, *p* < .001 for WFC X less than $75,000 X women), thereby supporting Hypothesis 4a.

Models 3a and 3b test the potential moderating effect of financial strain. The results indicate that the association between WFC and children’s problems does not vary across levels of financial strain. These patterns fail to support Hypothesis 2. We also test a three-way interaction term for gender differences in how financial strain might moderate the association between WFC and children’s problems. The results are statistically insignificant, thereby failing to support Hypothesis 4a. Nevertheless, there is clear evidence that financial strain is directly linked to elevated children’s problems for fathers (b = .067, *p* < .05) and mothers (b = .086, *p* <. 001) as shown in Models 1a and 1b.

In models 4a and 4b, we test the moderating effect of couple relationship dissatisfaction on the association between WFC and children’s problems. We observe that couple relationship dissatisfaction does not play a role of moderator in that association, which fails to support Hypothesis 3. We also test a three-way interaction term for gender differences in how couple relationship dissatisfaction might moderate the association between WFC and children’s problems. The results are statistically insignificant, which do not support Hypothesis 4b.

**Figure 2.**
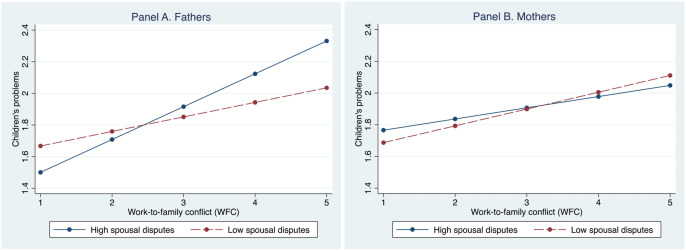
Predicted value in children’s problems by work-to-family conflict and spousal disputes for fathers (left) and mothers (right), with all covariates held at their means.

In Model 5a, we test whether spousal disputes moderate the association between fathers’ WFC and children’s problems. We find that spousal disputes amplify the positive association between fathers’ WFC and children’s problems (b = .116, *p* < .05). However, we do not find the same pattern for mothers (as shown in Model 5b). Figure 2 displays this interaction effect, with predicted values in children’s problems across WFC and spousal disputes for father and mothers, respectively. Together, these results partially support Hypothesis 3. Then, we test a three-way interaction term for gender differences in how spousal disputes moderate the association between WFC and children’s problems. We observe that the gender differences are marginally statistically significant, (b = −.138, *p* < .10 for WFC X spousal disputes X women), thereby supporting Hypothesis 4b.

## Discussion and Conclusion

A growing literature has explored the association between WFC and children’s outcomes (e.g., Matias et al., 2017; Yucel & Latshaw, 2020). However, given that stressors often coexist (Pearlin, 1989) and that experiencing multiple stressors simultaneously are often linked to greater health disparities (Young & Schieman, 2012), it is unclear about whether parents who experience WFC alongside with other household stressors are more detrimental to children’s well-being. To address this gap, we elaborated on the association between WFC and children’s problems with school, friends, and health by examining the potential contingencies in that process, specifically concentrating on different facets of household economic conditions and couple relationship quality. Like others (Matias et al., 2017; Yucel & Latshaw, 2020), we discovered that WFC was associated with greater children’s problems over time. However, we advance prior research by demonstrating this association was stronger for mothers in lower income households and for fathers who reported more frequent spousal disputes.

Our first set of findings align with the prediction of Bronfenbrenner’s (1986) ecological model of human development, suggesting that WFC is associated with greater children’s problems. To the best of our knowledge, there are no national longitudinal studies that estimate the relationship between WFC and children’s problems; one exception uses a measure that combines both directions of WFC and FWC (Dinh et al., 2017), which has potential limitations. This is an important contribution in light of recent concerns about whether the same adverse association between WFC and children’s problems can be replicated using longitudinal data (Matias et al., 2017). However, in separate analyses, we found little evidence that the association between WFC and children’s problems differed across gender. Some scholars have contended that gender ideology might matter more in shaping the consequences of WFC (Minnotte et al., 2010). For instance, Minnotte et al. (2010) discovered that the adverse effect of WFC was more detrimental to women’s marital satisfaction when they held more egalitarian gender ideologies. More attention to the possible moderating of gender versus gender ideologies is warranted.

After establishing the effect of WFC on children’s problems, our analyses revealed the moderating function of household income, suggesting that the adverse association between WFC and children’s problems was only stronger among mothers with lower household income and the gender differences were statistically significant. This pattern is partly consistent with the prediction of stress amplification theory (Young & Schieman, 2012), suggesting that the combination of lower family income and WFC would be even more detrimental for producing children’s problems. That is, lower household income strengthens the likelihood that WFC is viewed as a threat, which, in turn, amplifies the problematic family functioning and parenting practice. Moreover, the gendered pattern is consistent with the idea that breadwinner status continues to be an important characteristic of heterosexual men’s identities (Thébaud, 2010). Having a lower household income might threaten men’s masculinity, which encourages them not to engage in feminine activities, such as housework and childcare (Thébaud, 2010). Consequently, women take on more household tasks, which has been found to be detrimental to their mental health (Khawaja & Habib, 2007; Ruppanner et al., 2018) and ultimately adversely influence mother–child interactions. Based on these theoretical and empirical findings, we tested whether the division of housework and the division of childcare might explain the gendered moderating effect of household income on the association between WFC and children’s problems (i.e., WFC X household income X women). We found little evidence supporting these mediating mechanisms (results not shown but available upon request).

Nevertheless, the gendered pattern also aligns with prior research that women in households with low income are solely responsible for financial-related tasks (Thorne, 2010).^3^ For instance, there is evidence that men in households with low income often provide excuses (e.g., busy at work) to avoid helping their wives allocate money or negotiate with debt collectors (Thorne, 2010). Thus, due to a lack of support, women might struggle dealing with financial chores and thereby suffer severe emotional stress (Thorne, 2010). The detrimental effect of mother’s WFC might therefore be more consequential to children’s well-being due to the adverse mother–child interactions attributed to mothers’ greater stress in lower income households. Future studies should explore whether the division of financial-related tasks might account for these gendered observations.

Another central contribution of our study is the discovery of the moderating effect of the spousal disputes, suggesting that spousal disputes exacerbated the positive association between WFC and children’s problems among fathers but not mothers and that the gender differences were (marginally) significant. Partially consistent with the prediction of stress amplification theory (Young & Schieman, 2012), the adverse emotional and behavioral consequences attributed to spousal disputes might prevent parents from providing sufficient attention and coordination of care that negatively influence the well-being of their children, and the consequences might be even more acute when combined with WFC. However, one way to understand this gendered pattern involves research in the sociological literature on social support (Umberson & Thomeer, 2020). That is, according to the gender-role socialization perspective, women are more likely than men to develop more extensive social networks where they can access greater social support such as emotional care and comfort (Jung, 2014; Umberson & Thomeer, 2020). Thus, in addition to spouses, women rely on their nonmarital social support when dealing with relationship strains. By contrast, men are expected to be independent even when dealing with personal struggles because of masculine identity rooted in traditional gender norms (Bennett, 2007). In addition, research shows that men tend to view their spouses as a primary source of support (Donato et al., 2018). Thus, due to a lack of social support, men might adopt other coping strategies. For instance, there is evidence that spousal disputes are associated with anger (Doh et al., 2012) and that men and women respond to anger differently, with women being more likely to manage their anger by talking to someone and men being more likely to manage anger by having a drink or pill (Simon & Lively, 2010). It is therefore reasonable to assume that the adverse effect of spousal disputes on children’s problems might be more detrimental among fathers because of how they “cope” with disputes with their partners, which might negatively affect how they interact with the children. Nevertheless, these ideas are speculative, so more attention to the possible emotion-based explanations is warranted.

Although we hypothesized that the positive association between WFC and children’s problems would be stronger for households with greater financial strain, our results did not support this claim. We suspect that this nonsignificant pattern might be attributable to how financial strain was measured in our study. That is, financial strain might only have a significant effect on the association between WFC and children’s problems when it exceeds a certain threshold. Therefore, we reran the same set of analysis, but recoding the continuous measure of financial strain to a dummy variable (1 = high financial strain and 0 = low financial strain). We found that the same nonsignificant pattern remained (results not shown, but available upon request). Another potential explanation involves a conceptual aspect of financial strain. Research has suggested that the detrimental effect of financial strain is more pronounced among families in the middle/upper class (McCloud & Dwyer, 2011). For instance, people might experience financial strain because of high levels of spending, such as purchasing a house; or alternatively, due to economic downsizing, individuals might experience financial strain because of sudden losses of income (McCloud & Dwyer, 2011). Therefore, given that about 47.5% of parents work in non-managerial/professional occupations and that we have excluded unemployed individuals from our analyses, the moderating effect of financial strain might be undermined.

In a similar vein, we did not find evidence to support the moderating effect of marital dissatisfaction. We suspect that this nonsignificant pattern might be attributable to how marital dissatisfaction was measured in our study; that is, the moderating effect of marital dissatisfaction on the association between WFC and children’s problems might not be linear. To test this possibility, we performed additional analyses that recoded the continuous measure of marital dissatisfaction to a dummy variable (1= high marital dissatisfaction and 0= low marital dissatisfaction). We found that the same nonsignificant pattern remained (results not shown, but available upon request). Given that marital dissatisfaction typically focuses on emotional perceptions about one’s imitate relationship, we suspect that its detrimental effect might not be as harmful as behavioral responses (e.g., spousal disputes).

Before concluding, we wish to acknowledge five limitations of the present study. First, claims about causal ordering are not definitive. Based on prior theory and empirical evidence, we have made the case that WFC might generate children’s problems. However, it could be that children’s problematic outcomes influence parent–child interactions or/and parenting practices and FWC, which, in turn, contributes to greater WFC. Although we acknowledge that cross-lagged models might be a better methodological approach to document reverse causality (Maxwell & Cole, 2007), given our primary interest of examining moderating effects, the use of random effects models is more intuitive, which also allows us to capture the association between WFC and children problems over time, providing evidence that has additional precision compared to prior findings using cross-sectional data.

Second, these data are from individuals so we were unable to assess couple-level effects that might capture the interaction effects between maternal and paternal WFC on children’s problems (Hess & Pollmann-Schult, 2020). For instance, to reiterate, we only asked the respondents to report their WFC levels in our survey. However, it is possible that the WFC level reported by a respondent differs from the one that is perceived by his/her partner. Thus, this inconsistency might create spousal disputes or/and marital dissatisfaction, which could potentially negatively affect parenting practices and ultimately undermine children’s well-being. Nevertheless, our results do partially align with several pieces that employ couple-level data (Matias et al., 2017). Given that the association between WFC and children’s well-being might depend on how children’s outcomes are measured, future studies should replicate our findings by using similar measures of children’s problems along with incorporating couple-level data.

Third, scholars have highlighted the importance of the experiences of different stages of child development in evaluating the association between WFC and children’s well-being. For instance, compared to preschool-aged children, school-aged children are old enough to interact with parents in ways that provide their own accounts of family dynamics (Matias & Recharte, 2020). Given that children might have different problematic outcomes depending on their developmental stage (Campbell, 2006), future studies should explore the extent to which children’s ages moderate the association between parental WFC and children’s outcomes.

Fourth, we recognize that the alpha for children’s problems measure (0.62) as a potential concern. However, recent research shows that an alpha of 0.6–0.7 suggests an acceptable level of reliability (Ursachi, Horodnic, & Zait, 2015). Similar measures of children’s problems have been used in other recently published studies (e.g., Badawy & Schieman, 2020a). The relatively lower reliability of the measure likely indicates that our findings are somewhat conservative. We encourage future studies to enhance this measure in ways that might improve reliability. For instance, qualitative insights on this topic would be quite useful to provide more contextual meaning.

Fifth and finally, we recognize the relatively lower R-squared values reported in our study as a potential limitation, which were due to (mostly) nonsignificant effects of education, occupation, respondent’s work hours, and spousal’s work hours. Nevertheless, our results based on focal variables are still meaningful.

Despite these limitations, our findings from a unique longitudinal study of dual-earner households with dependent children offer new insights about household income and spousal disputes as moderators in the association between WFC and children’s problems. Our results find additional support to the usefulness of ecological model of human development in understanding how family functioning and parental practice permits the adverse effect of WFC to crossover to children’s well-being—and we integrate these ideas with other conceptual ideas like stress amplification theory.

## Notes

1. A reviewer commented that the bioecological model of human development (Bronfenbrenner & Morris, 2006) would provide support for the moderating effects discussed in our article. We explored more of this literature based on the reviewer’s suggestions. We agree that the bioecological model described by Bronfenbrenner and Morris (2006) is relevant and useful for our study. Their model highlights the interactive relationships among four main components, including Process, Person, Context, and Time (p. 795). Bronfenbrenner and Morris stressed that “[t]he *combination* of Person and Context exhibit a mutually reinforcing, multiplicative, indirect effect on the power of proximal processes as the “engines of development.” (p. 801) Unfortunately, however, we are unable to capture any of the Person (children) characteristics in our study, which limits our capacity to fully utilize the model. Here, we would like to emphasize the reviewers’ point that the bioecological model has the potential to provide support for examining the moderating mechanisms embedded within the family context and deserve more attention in the future studies.

2. To conduct random effect regression models, we had to reshape the dataset from a wide form to a long form. The sample size is often referred as “person-years” when the analyses are based on a long form. For instance, in our case, if an individual participates in all four waves of the study, we could say that this individual has four person-years.

3. We recognize that labeling this group as “low income” might miss important nuances and variations within this group, especially at the lower end of the income scale. But, as we noted earlier, small cell sizes necessitated the combining of cases into this broader income range.
